# An integrative analysis of tissue-specific transcriptomic and metabolomic responses to short-term dietary methionine restriction in mice

**DOI:** 10.1371/journal.pone.0177513

**Published:** 2017-05-16

**Authors:** Sujoy Ghosh, Laura A. Forney, Desiree Wanders, Kirsten P. Stone, Thomas W. Gettys

**Affiliations:** 1 Laboratory of Computational Biology, Pennington Biomedical Research Center, Baton Rouge, LA, United States of America; 2 Laboratory of Nutrient Sensing and Adipocyte Signaling, Pennington Biomedical Research Center, Baton Rouge, LA, United States of America; 3 Program in Cardiovascular & Metabolic Disorders and Centre for Computational Biology, Duke-NUS Graduate Medical School, Singapore; 4 Department of Nutrition, Georgia State University, Atlanta, GA, United States of America; INRA, FRANCE

## Abstract

Dietary methionine restriction (MR) produces a coordinated series of transcriptional responses in peripheral tissues that limit fat accretion, remodel lipid metabolism in liver and adipose tissue, and improve overall insulin sensitivity. Hepatic sensing of reduced methionine leads to induction and release of fibroblast growth factor 21 (FGF21), which acts centrally to increase sympathetic tone and activate thermogenesis in adipose tissue. FGF21 also has direct effects in adipose to enhance glucose uptake and oxidation. However, an understanding of how the liver senses and translates reduced dietary methionine into these transcriptional programs remains elusive. A comprehensive systems biology approach integrating transcriptomic and metabolomic readouts in MR-treated mice confirmed that three interconnected mechanisms (fatty acid transport and oxidation, tricarboxylic acid cycle, and oxidative phosphorylation) were activated in MR-treated inguinal adipose tissue. In contrast, the effects of MR in liver involved up-regulation of anti-oxidant responses driven by the nuclear factor, erythroid 2 like 2 transcription factor, NFE2L2. Metabolomic analysis provided evidence for redox imbalance, stemming from large reductions in the master anti-oxidant molecule glutathione coupled with disproportionate increases in ophthalmate and its precursors, glutamate and 2-aminobutyrate. Thus, cysteine and its downstream product, glutathione, emerge as key early hepatic signaling molecules linking dietary MR to its metabolic phenotype.

## Introduction

Restriction of dietary methionine intake by 80% produces a coordinated series of transcriptional, endocrine, and biochemical changes across multiple tissues, but the underlying mechanisms linking methionine restriction (MR) to its metabolic phenotype are poorly understood. The initial sensing of methionine is thought to occur in liver, where within 6 hours of introduction of the MR diet, increased transcription of the fibroblast growth factor 21 (*Fgf21*) gene produces an 8-fold increase in serum FGF21 [1]. Loss of function approaches indicate that FGF21 produces a combination of direct and indirect responses in adipose tissue that enhance the capacity of both brown and white adipose tissue to take up and oxidize glucose and fatty acids [2–4]. FGF21 works directly in adipocytes through receptor-mediated signaling to enhance both basal and insulin-dependent glucose uptake [5]. FGF21 also acts centrally to increase SNS-dependent remodeling and activation of thermogenesis in adipose tissue [3]. Thus, the MR-dependent increase in hepatic FGF21 provides a key link between the sensing of reduced dietary methionine in the liver and the translation of that sensing event into physiological responses [1, 4].

Although FGF21 appears to be a key mediator of a number of the indirect effects of MR in adipose tissue, the transcriptional effects of the diet in liver are most likely linked to MR-dependent effects on sulfur amino acid metabolism. These mechanisms remain poorly understood although recent work has argued that the downstream metabolite, glutathione has a previously unappreciated role in both hepatic insulin signaling [6] and protein kinase R-like endoplasmic reticulum kinase (PERK) signaling [1]. In the present study, a systems biology approach has been used to conduct an integrated analysis of the transcriptome and metabolome in liver and other affected tissues after short-term dietary MR. The goal is to identify important new transcriptional targets and biochemical processes that provide insights into the sensing and signaling mechanisms being engaged by the diet. The progression of our analyses is presented sequentially. First we report on differentially expressed genes and pathways responsive to MR by considering the transcriptome data from different tissues. This is followed by an independent investigation of the metabolomic data. Finally, we provide an integrated analysis of the transcriptomic and metabolomic datasets. Since all biological processes queried by the transcriptome do not have metabolomic measurements (e.g. inflammation-related pathways), and many metabolomic outcomes arise from post-transcriptional processes, this strategy ensures a more comprehensive assessment of the molecular landscapes and provides an excellent discovery platform to guide future studies.

## Materials and methods

### Animals and diets

All vertebrate animal experiments were reviewed and approved by the Pennington Institutional Animal Care and Use Committee using guidelines established by the National Research Council, the Animal Welfare Act, and the PHS Policy on humane care and use of laboratory animals. Twelve male C57BL/6J mice obtained from Jackson Labs (Bar Harbor, ME, USA) at 4 wks of age, individually housed in plastic cages with corncob bedding, and adapted to the control diet for 7 days prior to randomization to either the Control diet or the methionine-restricted (MR) diet for an additional 10–12 weeks. Details of the feeding paradigm and diets have been described previously [7], with the control diet containing 0.86% methionine, and the MR diet containing 0.17% methionine, and both diets containing no cysteine. The diets were formulated as extruded pellets and the energy content of both Control and MR diets was 15.96 kJ/g, with 18.9% of energy coming from fat (corn oil), 64.9% carbohydrate, and 14.8% from a custom mixture of L-amino acids. Diets and water were provided ad libitum, lights were on from 7 AM to 7 PM, and mice were housed at 23°C. The mice were euthanized at 11 AM using CO_2_-induced narcosis after a 4 h fast that began at 7 AM, and tissues were rapidly harvested and snap frozen in liquid nitrogen. Care was taken to minimize the numbers of animals used in this experiment in accordance with the ARRIVE guidelines (http://www.nc3rs.org.uk/page.asp?id=1357; see S1 ARRIVE Checklist).

### Isolation and analysis of RNA

Total RNA was isolated from inguinal white adipose tissue (IWAT), brown adipose tissue (BAT), liver, and skeletal muscle using RNeasy Mini Kits (QIAGEN, Valencia, CA). Concentration and integrity of the extracted RNA were assessed using a NanoDrop ND-1000 spectrophotometer (Nanodrop Technologies, Wilmington, DE) and an Agilent 2100 Bioanalyzer (Agilent Technologies, Forth Worth, TX). The RNA integrity number (RIN) for all samples ranged from 8.8 to 9.1. The gene expression profiles were assessed from 6 replicates for each tissue for each dietary group. cDNA libraries were prepared from the extracted RNA using the Applied Biosystems (AB) SOLiDSAGE kit, according to AB's instructions for the SOLiD 4 System. Gene expression profiling was performed by expression tag sequencing (35bp read length) on an AB SOLiD 5500XL sequencer. Sequence reads were mapped to the mouse reference genome (RefSeq RNA, mm9) using SOLiDSAGE, and further quantified to generate count data for each gene.

### SageSeq data processing and analysis

For each tissue, genes with minimum tag count ≥ 20 in at least one sample were retained for further analysis, resulting in 14394, 14102, 13745 and 14244 genes for IWAT, BAT, liver, and muscle, respectively. The expression profiling data has been deposited in NCBI under GEO accession GSE92463. A principal components analysis was performed to identify sample outliers usingPartek Genomics Suite, version 6.6. (Partek Inc., St. Louis, MO).

### Pathway enrichment analysis for transcriptomic data

Pathway enrichment analysis was conducted via gene-set enrichment analysis (GSEA) and over-representation analysis (ORA)-based approaches. For each tissue, GSEA was performed on the DESeq2 normalized expression signals via a running-sum statistic procedure [8,9] to determine the enrichment of *a priori* defined biological pathways from the Kyoto Encyclopedia of Genes and Genomes (KEGG) repository [10], obtained from the Molecular Signature Database (MSigDB)[11]. Statistical significance of pathway enrichment was ascertained by permutation testing over size-matched random gene-sets. Adjustments for multiple testing were performed via control of the FDR [12]. Overlap between significant pathways were visualized via the EnrichmentMap Cytoscape plugin [13] using the following filters–pathway p-value ≤ 0.005, q-value ≤ 0.1, overlap ≥ 50%.

ORA was conducted using Qiagen’s Ingenuity Pathway Analysis (IPA) tool (Qiagen, USA) on differentially expressed genes with p < 0.01 and absolute fold-change ≥ 1.5-fold, for each tissue. Statistical significance of over-represented pathways was ascertained via Fisher's exact test and adjusted for multiple testing via the FDR according to Benjamin-Hochberg [8].

### Analysis of upstream activators

An exploratory analysis was carried out in IPA to predict candidate upstream regulators (e.g transcription factors) whose activation/inhibition would be consistent with the observed changes in gene expression patterns. Genes were assigned to upstream regulators based on a curation of the literature in the Ingenuity Knowledge Base, and the expected effects of the regulator on its target gene’s expression was compared against the observed direction of gene expression change in the study. An ORA (Fisher’s exact test) was performed to determine whether a regulator was significantly enriched for differential expression of its target genes. The overall activation/inhibition status of the regulator was then inferred from the level of consistency in the observed up- or down-regulation of its target genes. The strength of evidence was statistically represented via a z-score, and regulators with an absolute z-score ≥ 2 were predicted to be ‘activated’ or ‘inhibited’, based on the sign of the z-score (http://pages.ingenuity.com/rs/ingenuity/images/0812%20downstream_effects_analysis_whitepaper.pdf).

### Metabolomic data analysis

A portion of IWAT, liver, and skeletal muscle from mice fed the respective control and MR diets was sent to Metabolon (Durham, NC, USA) for metabolomics analysis using GC/MS and UPLC-MS/MS analytical platforms. The quality control analysis included several technical replicate samples that were created from a homogeneous pool containing a small amount of all study samples. Instrument and process variability met Metabolon’s acceptance criteria and a total of 258, 359 and 290 metabolites were measured for IWAT, liver, and muscle samples, respectively (metabolites were not measured for BAT due to insufficient tissue amount). Metabolite data on each sample was normalized to unit median and analyzed for significant differences between the control and MR groups via Welch’s test [14]. The level of false positives was controlled by FDR [12]. Pathway enrichment analysis from metabolite data in each tissue was conducted via the Metabolite Biological Role tool [15] by querying KEGG pathways and using metabolites with a q-value ≤ 20%, as input. Significantly enriched pathways were visualized by the KEGG Mapper tool (http://www.genome.jp/kegg/mapper.html).

## Results

### Principal components analysis

**Fig 1:**
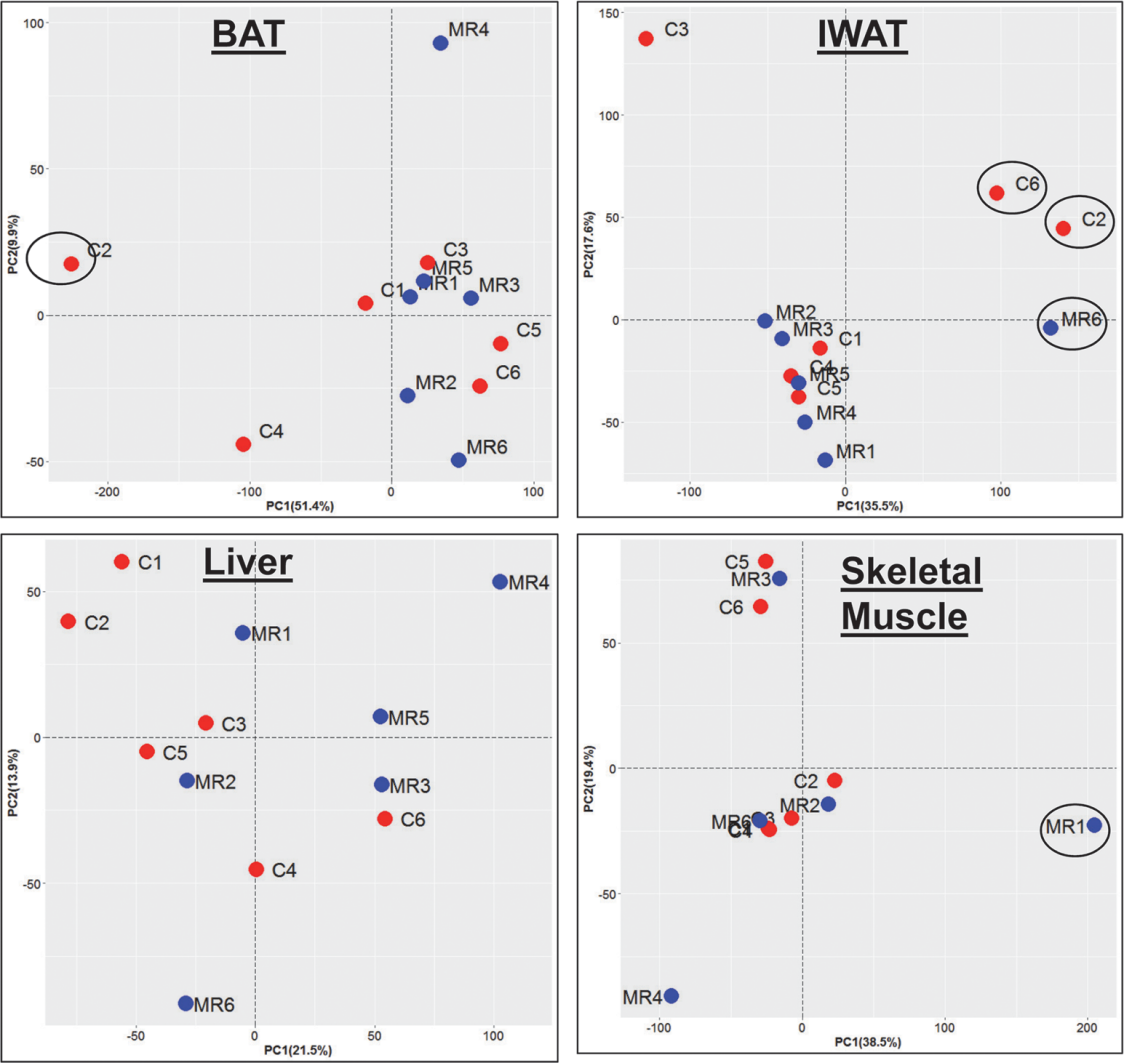
Principal components analysis on SAGE data. PCA was performed on DESeq2 generated signals from IWAT, liver, BAT and skeletal muscle after retaining genes with minimum tag count ≥20 (≥18 for muscle) in at least one sample in each tissue. The first two principal components are plotted, and the proportion of variance explained by each component is indicated on the respective axis titles. Control samples are shown in red and MR-treated samples are shown in blue. The circled samples represent outliers and were removed from further analysis.

A principal components analysis was performed to identify sample outliers and identified three outlier samples for IWAT and one outlier sample each for BAT and muscle (Fig 1). After excluding outliers, the remaining samples were subjected to data normalization and differential gene expression analysis using the DESeq2 tool. Prior to DESeq2 analysis, all signals < 1 were thresholded to 1. The significance of differential expression was ascertained by modeling the mean-variance relationship through the negative binomial distribution, and by adjustments for multiple testing via the false discovery rate (FDR) [8].

### Analysis of the transcriptome

**Fig 2:**
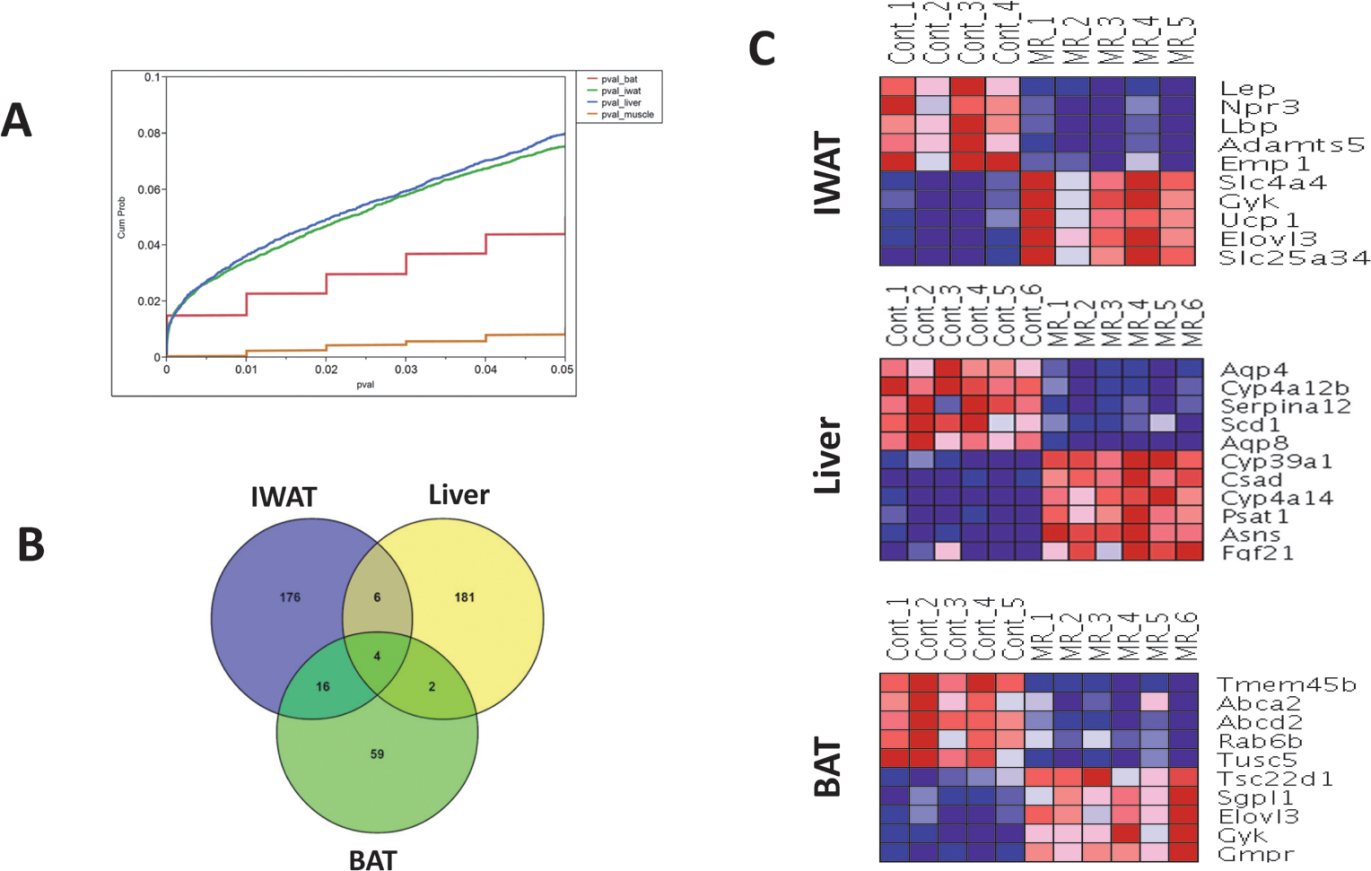
Overview of differential gene expression in tissues. (A) Cumulative probability plot of differentially expressed genes in IWAT (green), liver (blue), BAT (red) and skeletal muscle (brown), depicting the fraction of all genes (y-axis) satisfying the various nominal p-value cutoffs (x-axis). Only genes with nominal p-value ≤0.05 are shown. (B) Overlap analysis of the differentially expressed genes, with adjusted p-value ≤0.05, in IWAT, liver and BAT (no genes identified at this threshold in skeletal muscle). (C) Heatmaps depicting expression patterns of top 10 differentially expressed genes (5 up-regulated and 5 down-regulated) across methionine-restricted and Control samples in IWAT, liver and BAT. Genes are selected based on the magnitude of fold-changes, and all genes have an adjusted p-value ≤0.05. Expression values are row-normalized for each gene with shades of blue indicating lower expression and red indicating higher expression.

We first determined the extent of differential gene expression induced by MR treatment in BAT, IWAT, liver, and skeletal muscle by comparing the proportion of total genes that were significantly differentially expressed at different nominal p-value cutoffs. Results are shown as a cumulative probability plot in Fig 2A. Larger proportions of differentially-expressed genes were observed in liver and IWAT at all p-value cutoffs, finally reaching 7.9% and 7.5% of all genes at p = 0.05, respectively. In contrast, the overall extent of differentially-expressed genes was moderate in BAT (4.7%) and very low in muscle (0.9%). After controlling for false discoveries (FDR ≤ 5%), there were no significantly differentially expressed genes in muscle. In Fig 2B, we compared the overlap among differentially-expressed genes (FDR ≤ 5%) from IWAT, liver, and BAT. The overlap among the lists was minimal, suggesting that the transcriptomic responses to MR were tissue-specific. The top 10 genes showing the largest fold-changes in MR vs. Control (FDR < 5% for all genes) are shown in Fig 2C (additional gene results are shown in S1 Table). As reported previously [6], hepatic FGF21 was induced by dietary MR and has been included in the liver-specific heatmap.

**Fig 3:**
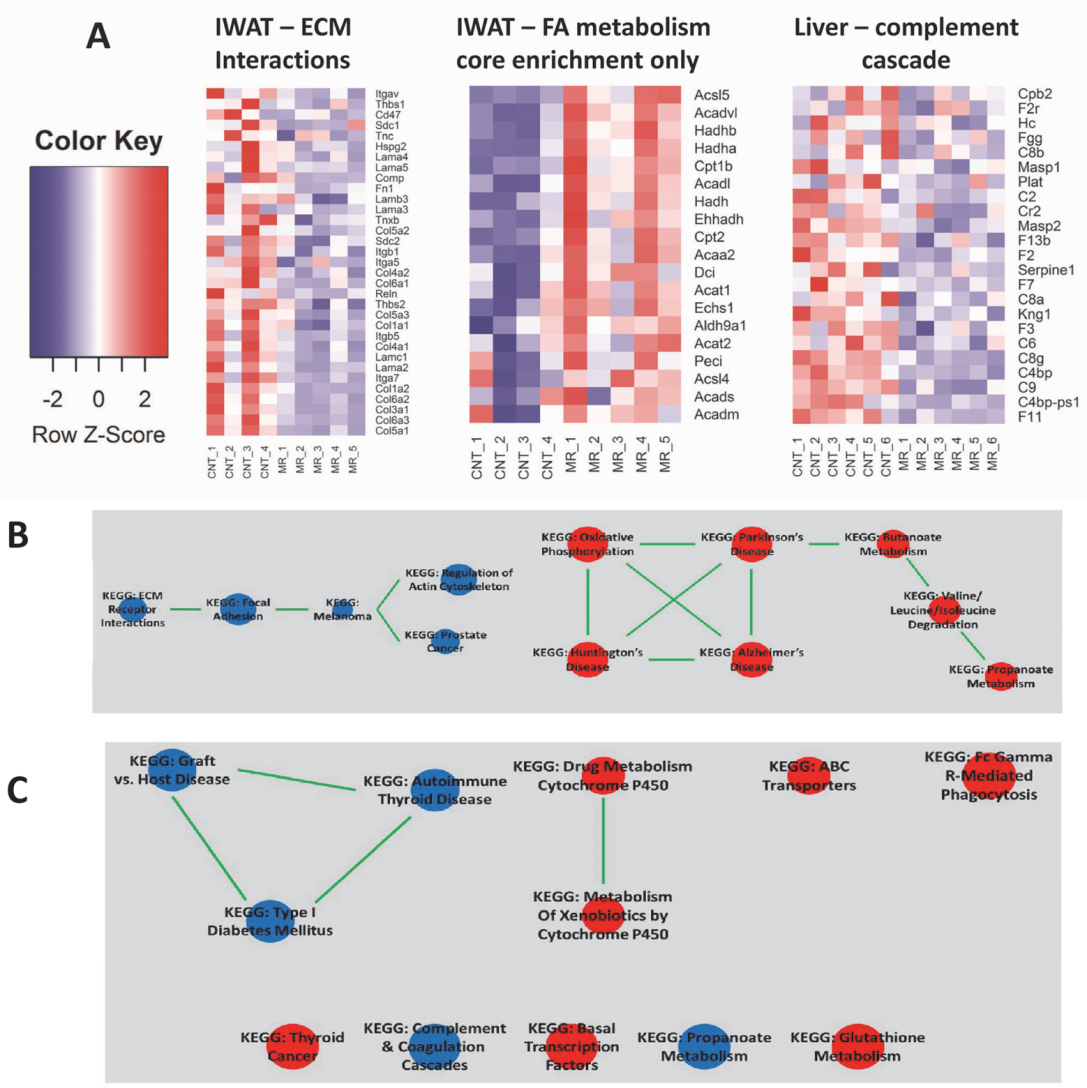
Pathway enrichment analysis based on transcriptomic response to MR. (A) Examples of top-scoring KEGG pathways in IWAT and liver, identified by gene-set enrichment analysis (GSEA). The heatmaps show expression patterns for the genes contributing to core enrichment of ‘Fatty acid metabolism’ pathway in IWAT and ‘Complement cascade’ pathway in liver (blue and red indicated lower and higher expression levels, respectively. (B) Gene overlap among top-scoring pathways in IWAT. Up-regulated and down-regulated pathways in MR are shown in red and blue respectively. Pathways sharing >50% of their genes are connected via a green edge. (C) Overlap among top-scoring pathways based on gene expression data in liver. GSEA significant pathways (FDR<0.1) were analyzed via the Enrichment Map application in Cytoscape (v3.4.0) for identifying pathways with >50% overlap in their component genes. Pathways upregulated in MR-treated liver samples are shown in red and downregulated pathways are shown in blue.

To obtain a more comprehensive picture of the biological mechanisms affected by MR, we carried out gene-set enrichment analysis (GSEA) of transcriptomic data from IWAT, liver, BAT, and muscle. The top-scoring pathways in each tissue (FDR ≤ 5% for IWAT and ≤ 10% for liver, BAT and muscle) are shown in Table 1. Notably, at a FDR cutoff of 10%, no pathways were found to be significantly up-regulated by MR in liver or muscle, or significantly down-regulated in BAT (complete results available in S2 Table). In IWAT, we observed a general up-regulation of metabolic pathways and down-regulation of processes related to cytoskeletal matrix organization, as indicated respectively by the ‘ECM receptor interactions’ and ‘fatty acid metabolism’ gene-sets in Fig 3A. In contrast, the main biological signals from the MR liver transcriptome implied a down-regulation of inflammation-related pathways, as illustrated by the ‘complement cascade’ pathway. We then interrogated the extent of gene overlap among the top regulated pathways in IWAT and liver (FDR ≤ 5%). In Fig 3B, IWAT up- and down-regulated pathways with ≥ 50% overlap are shown, which clearly illustrates the compositional redundancies of ‘Huntington’s disease’, ‘Parkinson’s disease’, and ‘Alzheimer’s disease’ pathways with the more mechanistically informative ‘oxidative phosphorylation’ pathway. A similar analysis on the liver-related significant pathways is presented in Fig 3C.

**Table 1:** Key transcriptionally affected KEGG pathways in MR treated tissues.

NAME	SIZE	NES	NOM p-val	FDR q-val	Direction	Tissue
TYPE_I_DIABETES_MELLITUS	20	-1.860	0.000	0.040	Dn_MR	Liver
COMPLEMENT_AND_COAGULATION_CASCADES	60	-1.872	0.002	0.070	Dn_MR	Liver
PROPANOATE_METABOLISM	28	-1.717	0.010	0.078	Dn_MR	Liver
GRAFT_VERSUS_HOST_DISEASE	17	-1.751	0.002	0.091	Dn_MR	Liver
AUTOIMMUNE_THYROID_DISEASE	16	-1.721	0.006	0.095	Dn_MR	Liver
STEROID_BIOSYNTHESIS	15	2.003	0.000	0.016	Up_MR	BAT
ECM_RECEPTOR_INTERACTION	70	-2.310	0.000	0.000	Dn_MR	Muscle
PROPANOATE_METABOLISM	29	-1.861	0.002	0.059	Dn_MR	Muscle
FOCAL_ADHESION	171	-2.237	0.000	0.000	Dn_MR	IWAT
ECM_RECEPTOR_INTERACTION	63	-2.188	0.000	0.000	Dn_MR	IWAT
BASAL_CELL_CARCINOMA	39	-2.057	0.000	0.001	Dn_MR	IWAT
CYTOKINE_CYTOKINE_RECEPTOR_INTERACTION	161	-1.972	0.000	0.002	Dn_MR	IWAT
TIGHT_JUNCTION	101	-1.732	0.000	0.040	Dn_MR	IWAT
MELANOMA	54	-1.734	0.002	0.045	Dn_MR	IWAT
HYPERTROPHIC_CARDIOMYOPATHY_HCM	63	-1.743	0.004	0.048	Dn_MR	IWAT
REGULATION_OF_ACTIN_CYTOSKELETON	167	-1.704	0.000	0.050	Dn_MR	IWAT
OXIDATIVE_PHOSPHORYLATION	95	3.000	0.000	0.000	Up_MR	IWAT
PARKINSONS_DISEASE	92	2.934	0.000	0.000	Up_MR	IWAT
CITRATE_CYCLE_TCA_CYCLE	28	2.761	0.000	0.000	Up_MR	IWAT
HUNTINGTONS_DISEASE	143	2.676	0.000	0.000	Up_MR	IWAT
PYRUVATE_METABOLISM	34	2.545	0.000	0.000	Up_MR	IWAT
PROPANOATE_METABOLISM	29	2.508	0.000	0.000	Up_MR	IWAT
ALZHEIMERS_DISEASE	130	2.448	0.000	0.000	Up_MR	IWAT
GLYCOLYSIS_GLUCONEOGENESIS	49	2.348	0.000	0.000	Up_MR	IWAT
PEROXISOME	75	2.263	0.000	0.000	Up_MR	IWAT
VALINE_LEUCINE_AND_ISOLEUCINE_DEGRADATION	41	2.244	0.000	0.000	Up_MR	IWAT
FATTY_ACID_METABOLISM	36	2.220	0.000	0.000	Up_MR	IWAT
PENTOSE_PHOSPHATE_PATHWAY	22	2.137	0.000	0.000	Up_MR	IWAT
AMINOACYL_TRNA_BIOSYNTHESIS	40	2.036	0.000	0.001	Up_MR	IWAT
BUTANOATE_METABOLISM	28	1.972	0.000	0.002	Up_MR	IWAT
FRUCTOSE_AND_MANNOSE_METABOLISM	33	1.956	0.000	0.002	Up_MR	IWAT
CARDIAC_MUSCLE_CONTRACTION	52	1.841	0.000	0.007	Up_MR	IWAT
TERPENOID_BACKBONE_BIOSYNTHESIS	13	1.781	0.011	0.011	Up_MR	IWAT
LIMONENE_AND_PINENE_DEGRADATION	10	1.736	0.014	0.017	Up_MR	IWAT
PPAR_SIGNALING_PATHWAY	59	1.725	0.005	0.018	Up_MR	IWAT
BIOSYNTHESIS_OF_UNSATURATED_FATTY_ACIDS	19	1.719	0.000	0.019	Up_MR	IWAT
TRYPTOPHAN_METABOLISM	32	1.657	0.015	0.032	Up_MR	IWAT
GLYCEROPHOSPHOLIPID_METABOLISM	60	1.617	0.004	0.043	Up_MR	IWAT

Pathways were analyzed for enrichment in differentially expressed genes by GSEA method. Pathways with FDR ≤ 0.05 for IWAT and ≤ 0.1 for liver, BAT, muscle are shown. Col 1, name of KEGG pathway; col 2, pathway size (number of genes in pathway); col 3, Normalized Enrichment Score, a measure of pathway enrichment adjusted for pathway size; col 4, nominal p-value for pathway significance; col 5, pathway false discovery rate (expressed as q-value); col 6, direction of pathway enrichment in MR compared to Control; col 7, tissue of analysis.

**Fig 4:**
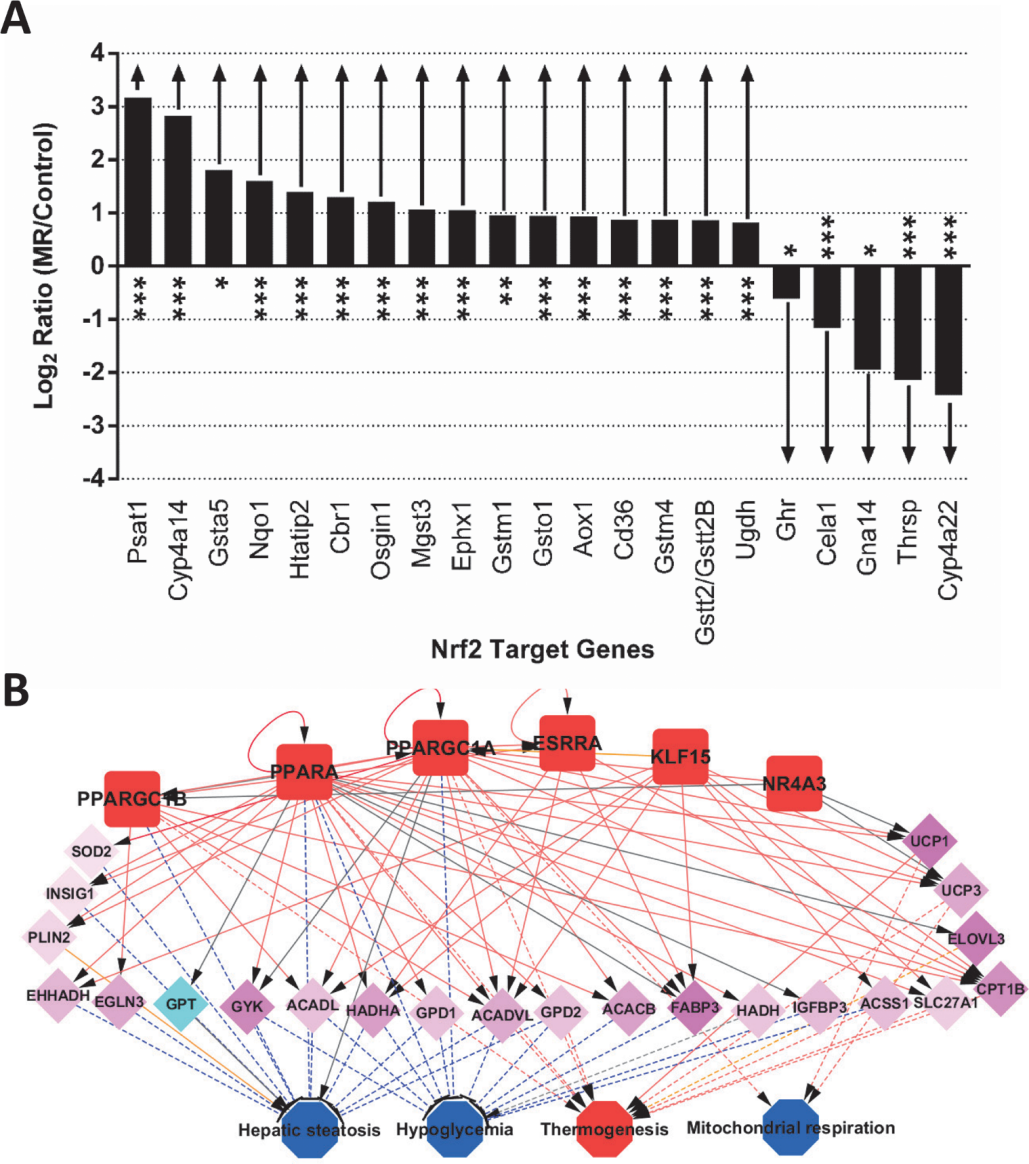
Prediction of upstream regulators’ involvement in the transcriptomic response. (A) Response of NFE2L2 target genes in liver from MR-fed mice, compared to Control. The list of NFE2L2 target genes was obtained from the Ingenuity Knowledge Base and their fold-change (MR vs. Control, log2 scale) is plotted on the y-axis. Statistical significance of differential expression is indicated for each gene—*, p<0.05; **, p<0.005; ***, p<0.0005. (B) Predicted transcription factor (TF) network in MR-treated IWAT. Several transcription factors with predicted activation from IPA analysis are integrated into a network based on overlapping target genes, and the effect of these changes on downstream processes are also modeled. Interactions between TF:target genes and between genes: downstream processes are shown as solid or dashed lines, respectively. Genes (middle panel) are color coded based on their observed upregulation or downregulation in MR vs. Control samples. Upstream regulators and downstream processes are colored based on their activation or inhibition status (red and blue, respectively). Edges representing activating interactions are shown in red, inhibitory interactions in blue, unknown effects in gray, and findings inconsistent with literature in yellow.

The gene expression changes following MR can be viewed as an integrated response, consequent to the activation or inhibition of upstream gene-regulators, such as transcription factors. To generate predictions on such activation or inhibition states of transcription factors that would be consistent with the observed transcriptomic changes, we interrogated the liver and IWAT transcriptome data via the Upstream Regulator analysis tool in Ingenuity Pathway Analysis [16]. The liver transcriptome changes were found to be consistent with the predicted regulation of several transcription factors including ATF4, NUPR1, PPARA, SP1, and NFE2L2 (predicted activation) and MLXIPL, CREBBP, SREBF2, HNF4A (predicted inhibition). Of these, one with the strongest evidence for regulation was the nuclear factor erythroid2-like 2, or NFE2L2 transcription factor (overlap p = 1.12e-13, z-score = 3.641). The expression profile of NFE2L2 target genes (Fig 4A) demonstrates a majority of such genes to have highly significant differences in mean expression between MR and Control samples (adjusted p ≤ 0.05 for 18/21 genes) with moderate to large effect sizes (absolute fold-changes 2-fold or higher). Notably, the observed directions of gene expression changes were always consistent with predictions based on the known regulation of these genes by NFE2L2; thus genes up-regulated by NFE2L2 activation were up-regulated in MR (e.g. *Psat1*, *Cyp4a14*) while genes up-regulated upon NFE2L2 inhibition were down-regulated (e.g. *Ghr*, *Gna14*). A similar upstream regulator analysis in IWAT identified several transcription factors related to nutrient metabolism, and further connected them into a broader gene regulatory network containing common gene targets and biological processes associated with such genes. The most notable IWAT-specific transcription factor-driven mechanistic networks is illustrated in Fig 4B, where several predicted to be activated transcription factors (PPARGC1B, PPARA, PPARGC1A, ESRRA, KLF15 and NR4A3), were interconnected through their regulation of a common repertoire of genes (middle layer of the network). The transcription factors and a subset of their target genes were further linked to downstream biological functions, based on mining of the IPA Knowledge Base, allowing for an exploration of the possible functional consequences of regulator-driven transcriptomic changes. Additional details of the upstream regulator analysis are provided in S3 Table.

### Analysis of the metabolome

**Fig 5:**
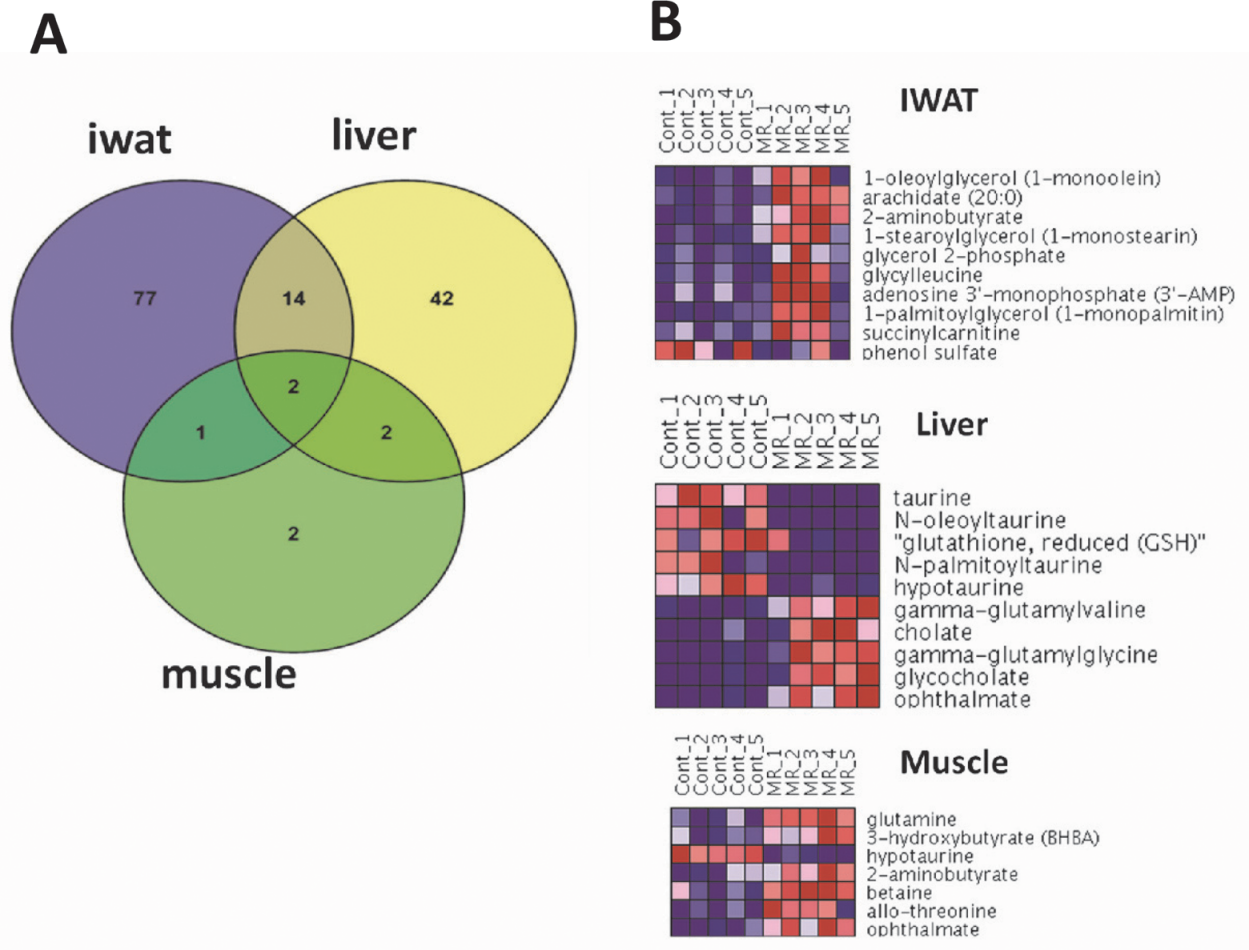
Overview of metabolite expression in tissues. (A) Overlap analysis of the differentially expressed metabolites, with adjusted p-value ≤0.05, in IWAT, liver and muscle. (B) Heatmaps depicting expression patterns of top differentially expressed metabolites across MR and Control samples in IWAT, liver and muscle. Metabolites are selected based on the magnitude of fold-changes, and all metabolites have an adjusted p-value ≤0.05 (for muscle, only 7 metabolites met the adjusted p-value criterion). Expression values are row-normalized for each metabolite with shades of blue indicating lower expression and red indicating higher expression.

Our strategy for the analysis of metabolomics data mirrored the approach taken for transcriptome analysis. First, we performed univariate statistical analysis to identify metabolites displaying significant responses to MR in IWAT, liver, and muscle after controlling for false discoveries. The number of significantly altered metabolites (FDR ≤ 5%) in each tissue and their inter-tissue overlap are depicted in Fig 5A. The largest number of metabolite changes were observed in IWAT (94 metabolites), followed by liver (60 metabolites) and then muscle with very few changes (7 metabolites). A total of 16 metabolites were identified as significantly altered in both IWAT and liver, but the overall overlap across all 3 tissues was negligible. The top 10 altered metabolites in each tissue (based on fold-changes) are shown in Fig 5B (the full list of metabolite expression in each tissue is presented in S4 Table).

We next sought to identify the KEGG metabolic pathways that were enriched for significantly differentially expressed metabolites in IWAT and liver. Results are summarized in Table 2 for pathways that were significant at the 5% FDR level. The largest number of significant pathways was observed in IWAT and predominantly involved pathways related to fatty acid and amino acid metabolism (e.g. *biosynthesis of unsaturated fatty acids*, FDR = 4.5e-09; *cysteine and methionine metabolism*, FDR = 9.5e-04). In liver, pathways related to ‘*primary bile acid biosynthesis*,*’* ‘*glycine-serine-threonine metabolism*,*’* and ‘*ABC transporters*’ displayed the most significant enrichment (FDR ≤ 0.0002).

**Table 2:** Key metabolically regulated KEGG pathways in MR treated tissues.

KEGG pathways	IWAT_neg log FDR	Liver_neg log FDR	Muscle_neg log FDR
Metabolic pathways	11.87	5.17	2.68
ABC transporters	8.87	3.34	3.45
Aminoacyl-tRNA biosynthesis	8.87	2.98	2.79
Biosynthesis of unsaturated fatty acids	8.34		1.10
beta-Alanine metabolism	4.92		
Glycine, serine and threonine metabolism	2.06	3.68	4.47
Pantothenate and CoA biosynthesis	4.46	2.90	1.61
Purine metabolism	4.46		
Glutathione metabolism	4.31	2.37	
Primary bile acid biosynthesis	1.68	3.68	
Arginine and proline metabolism	3.66	2.01	0.84
Pyrimidine metabolism	3.57	3.34	
Cyanoamino acid metabolism		3.20	
Taurine and hypotaurine metabolism	1.68		3.16
Cysteine and methionine metabolism	3.02		1.10
Alanine, aspartate, glutamate metabolism	2.99		
Histidine metabolism	2.29		
Fatty acid biosynthesis	2.06		
Sulfur metabolism	1.80		1.89
Butanoate metabolism	1.86		
Amino sugar, nucleotide sugar metabolism	1.80		
Renal cell carcinoma	1.76		
Glycerophospholipid metabolism	1.68		
Lysine degradation	1.68		
Riboflavin metabolism	1.68		
Lysosome	1.59		
Starch and sucrose metabolism	1.58		
Parkinson's disease	1.50		
Linoleic acid metabolism	1.42		
Nitrogen metabolism	1.42		
Galactose metabolism	1.38		
Valine, leucine and isoleucine biosynthesis	1.34		
Oxidative phosphorylation	1.31		

Pathways were analyzed for enrichment of differentially expressed metabolites upon MR exposure. Col 1, name of pathway; col 2–4, statistical evidence for pathway enrichment in IWAT, liver and muscle respectively (negative logarithm of the FDR). Only pathways containing ≥5 metabolites, and with a FDR<0.1 in at least one tissue are shown.

In addition to the above KEGG-based pathway analysis, we sought to compare the relative expression of metabolites in IWAT and liver that were classified by their compound classes. This analysis is distinct from that of the more mechanism-oriented compound lists assembled in KEGG. Analysis by compound class showed a strong difference in IWAT and liver expression for metabolites belonging to the long-chain fatty acid (LCFA), polyunsaturated fatty acid (PUFA) and gamma-glutamyl amino acid (GGAA) classes (Table 3). More specifically, all reported LCFA species were found to be significantly elevated in IWAT (p < 0.05), whereas most of the liver LCFA were downregulated by MR, with only 4/15 species reaching a significance level of p < 0.05. In a similar manner, the majority of PUFAs trended towards up-regulation in IWAT from MR-fed mice (p < 0.1), whereas PUFAs from MR livers were largely downregulated with no significant differences observed (p > 0.1). Conversely, large, statistically significant changes in gamma-glutamyl amino acids (GGAAs) were only observed in MR livers, whereas only 1/7 GGAAs could be detected in IWAT.

**Table 3:** Comparison of metabolite expression in IWAT and liver, based on metabolite class.

Compound	Compound Class	Ratio WT MR vs Con Liver	Ratio WT MR vs Con IWAT
10-heptadecenoate (17:1n7)	LCFA	-1.85	1.57
10-nonadecenoate (19:1n9)	LCFA	-1.59	2.24
arachidate (20:0)	LCFA	-1.22	3.02
cis-vaccenate (18:1n7)	LCFA	-1.96	1.67
eicosenoate (20:1n9 or 11)	LCFA	-2.08	2.05
erucate (22:1n9)	LCFA	-2.38	2.21
margarate (17:0)	LCFA	-1.16	1.61
myristate (14:0)	LCFA	-1.69	1.99
myristoleate (14:1n5)	LCFA	-1.43	1.73
nonadecanoate (19:0)	LCFA	-1.25	1.88
oleate (18:1n9)	LCFA	1.08	2.29
palmitate (16:0)	LCFA	-1.35	1.49
palmitoleate (16:1n7)	LCFA	-2.08	1.43
pentadecanoate (15:0)	LCFA	1.22	1.93
stearate (18:0)	LCFA	-1.14	1.79
adrenate (22:4n6)	PUFA	-1.45	1.19
arachidonate (20:4n6)	PUFA	-1.19	1.51
dihomo-linoleate (20:2n6)	PUFA	-1.43	2.13
dihomo-linolenate (20:3n3 or n6)	PUFA	-1.39	1.25
docosadienoate (22:2n6)	PUFA	-1.64	2.17
docosahexaenoate (DHA; 22:6n3)	PUFA	-1.04	1.42
docosapentaenoate (n3 DPA; 22:5n3)	PUFA	1.00	1.03
docosapentaenoate (n6 DPA; 22:5n6)	PUFA	1.11	1.44
eicosapentaenoate (EPA; 20:5n3)	PUFA	-1.59	0.92
linoleate (18:2n6)	PUFA	-1.14	1.64
linolenate (18:3n3 or 6)	PUFA	-1.09	1.58
stearidonate (18:4n3)	PUFA	1.16	ND
gamma-glutamylglycine	GGAA	13.10	ND
gamma-glutamylvaline	GGAA	7.37	ND
gamma-glutamylleucine	GGAA	7.03	1.54
gamma-glutamylthreonine*	GGAA	6.79	ND
gamma-glutamylisoleucine*	GGAA	4.24	ND
gamma-glutamylphenylalanine	GGAA	1.43	ND
gamma-glutamylglutamate	GGAA	1.26	ND
5-oxoproline	Sulfur metabolite	2.21	1.24
cysteine	Sulfur metabolite	2.39	1.48
cysteine-glutathione disulfide	Sulfur metabolite	3.01	1.36
cysteinylglycine	Sulfur metabolite	-1.29	ND
glutathione, oxidized (GSSG)	Sulfur metabolite	-1.16	1.47
glutathione, reduced (GSH)	Sulfur metabolite	-3.84	2.41
homocysteine	Sulfur metabolite	5.53	ND
hypotaurine	Sulfur metabolite	-2.94	-1.72
methionine	Sulfur metabolite	1.38	1.22
N-acetylmethionine	Sulfur metabolite	1.34	1.5
ophthalmate	Sulfur metabolite	16.25	1.46
S-adenosylhomocysteine (SAH)	Sulfur metabolite	1.15	1.82
S-adenosylmethionine (SAM)	Sulfur metabolite	1.03	ND
S-methylcysteine	Sulfur metabolite	1.06	ND
S-methylglutathione	Sulfur metabolite	-1.51	ND
taurine	Sulfur metabolite	-9.09	-1.07

Col 1, compound name; col 2, compound class; col 3, ratio of average expression in MR vs Control liver samples; col 4, ratio of average expression in MR vs Control IWAT samples. Expression ratios are shaded based on their statistical significance–p<0.05, dark gray; 0.05<p<0.1, light gray; p>0.1, unshaded. LCFA, long-chain fatty acid; PUFA, polyunsaturated fatty acid; GGAA, gamma-glutamyl amino acid.

### Integrated analysis of the transcriptome and metabolome

**Fig 6:**
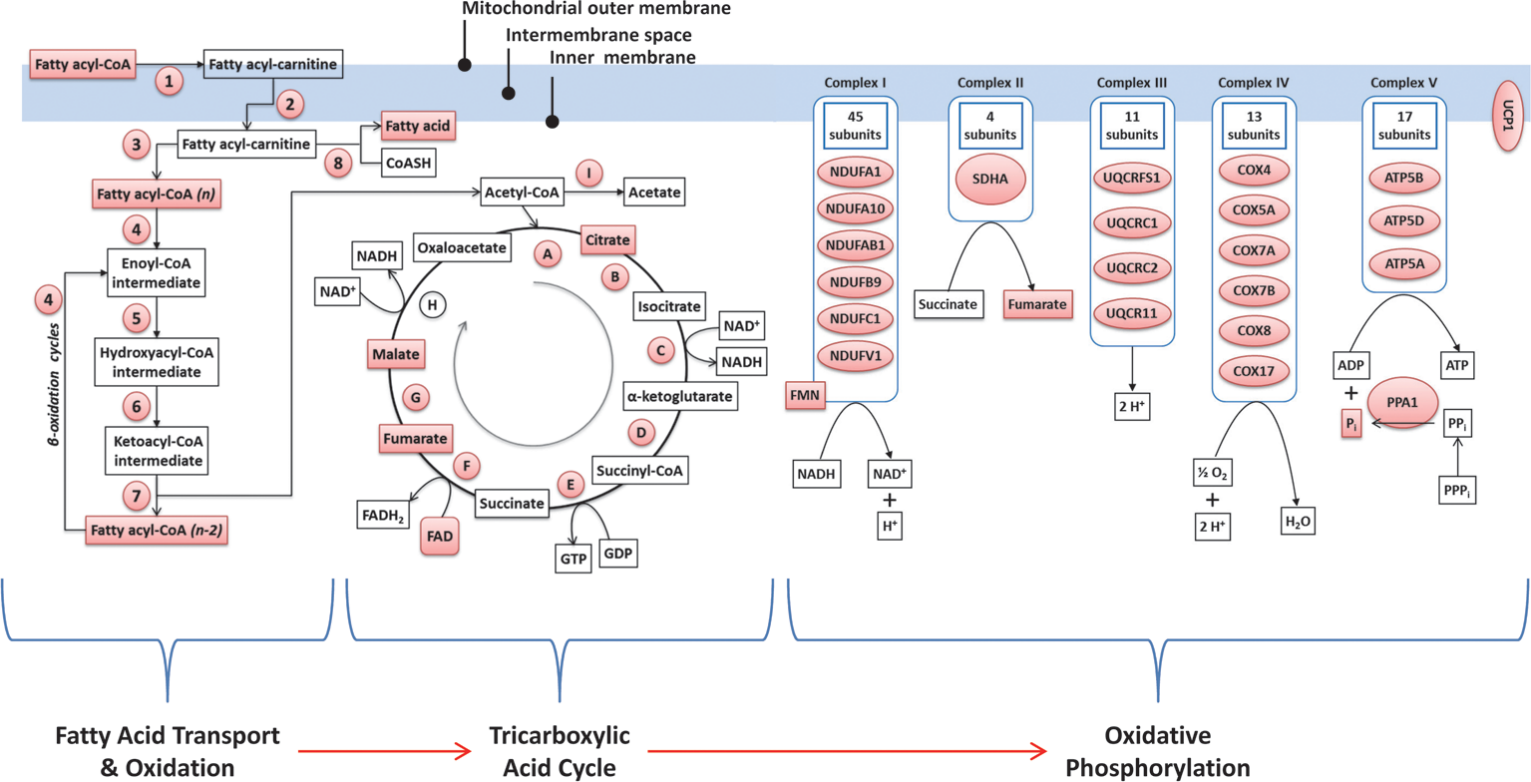
Integrated analysis of metabolite and transcript expression for pathways related to ‘fatty acid transport and metabolism, tricarboxylic acid cycle, and oxidative phosphorylation’ in MR IWAT compared to controls. The relationship between the 3 pathways are shown and genes/metabolites involved in each pathway are indicated either by their original names or by numeric and alphabetic proxies. Genes and metabolites that are significantly upregulated in MR IWAT compared to Control are shown in pink. The full names of all genes/metabolites, their fold-changes and estimates of statistical significance are detailed in Table 4.

**Fig 7:**
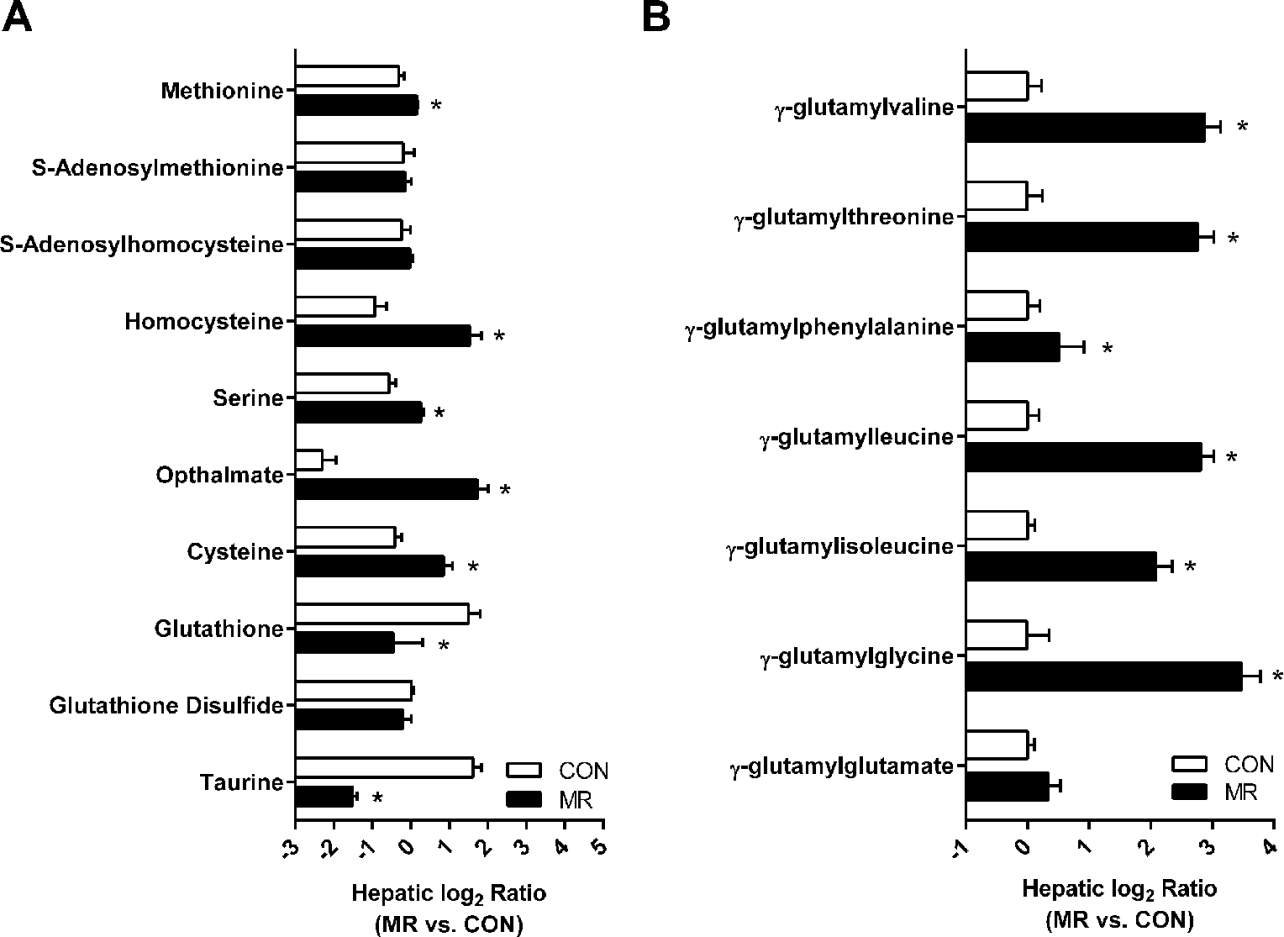
Analysis of sulfur-containing metabolites in MR treated liver. (A) Relative expression of metabolites measured on the methionine to cysteine transmethylation and transsulfuration pathways and (B) relative expression of gamma-glutamyl amino acids. * in (A) and (B) indicate difference between CON and MR at p < 0.05. (C).

**Fig 8:**
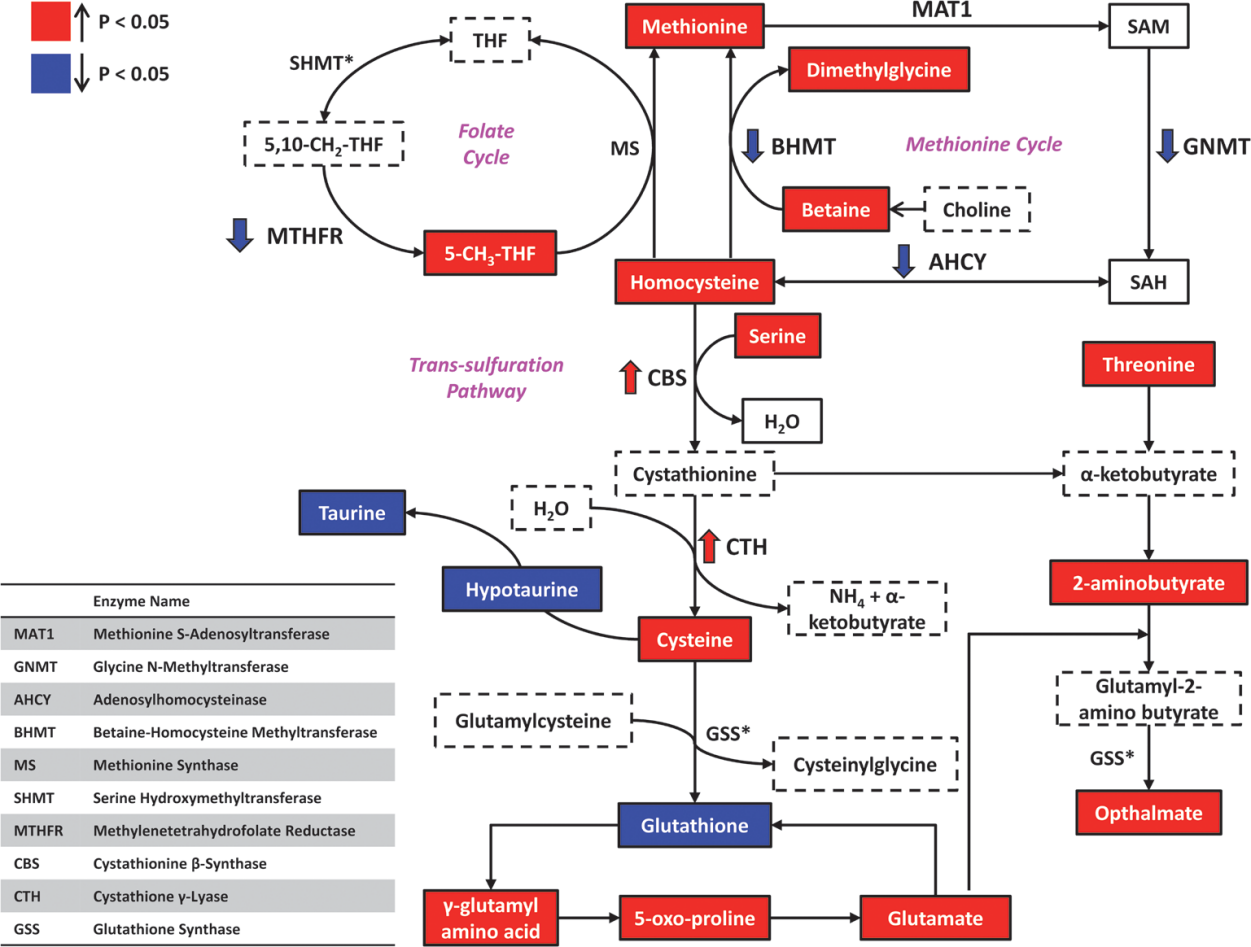
The effect of dietary MR on levels of key molecules and metabolites in hepatic sulfur amino acid metabolism. The changes in expression levels of key molecules in methionine, cysteine and glutathione metabolism are shown. Experimentally measured metabolites are shown as solid boxes, and experimentally measured genes are shown in bold. Dashed boxes refer to metabolites not measured. Upregulated metabolites are colored in red and downregulated metabolites are colored in deep blue (p < 0.05).

To generate additional insights from a combination of the transcriptome and the metabolome data, we carried out integrative analysis by focusing on genes and metabolites that were differentially regulated by MR (FDR ≤ 0.2). By jointly considering the transcriptomic and metabolomics data, we observed evidence of upregulation in highly interconnected biological processes involving mitochondrial fatty acid transport, fatty acid beta-oxidation, tricarboxylic acid cycle, and electron transport/oxidative phosphorylation (Fig 6 and Table 4). Similarly, an important finding from the joint transcriptome-metabolome analysis attributable to the MR diet was a significant liver-specific alteration in sulfur amino acid metabolite levels (Table 3). An integrated view of several metabolic processes involving sulfur-containing amino acid metabolites affected by dietary MR is depicted in Fig 7A and 7B and Fig 8.

**Table 4:** Gene and metabolite changes in fatty acid transport and metabolism, tricarboxylic acid cycle and oxidative phosphorylation pathways in MR-treated adipose tissue. The gene and metabolite identifiers used in Fig 6 are mapped to their real descriptions in this table.

GENES				METABOLITES			
ID in Fig 6	Name	Fold-change	FDR (q value)	ID in Fig 6	Name	Fold-change	FDR (q value)
**Fatty acid transport and metabolism pathway**							
1	Cpt1b	5.11	0.02	Fatty acyl CoA	myristate (14:0)	1.99	0.01
2	Slc25a20 (Cat)	2.89	1.30E-03		palmitate (16:0)	1.49	0.01
3	Cpt2	2.48	0.11		stearate (18:0)	1.79	8.00E-03
4	Acadl	2.45	0.01		oleate (18:1n9)	2.29	7.00E-03
	Acadvl	3.29	7.89E-05		myristate (14:0)	1.99	0.01
5	Hadha	3.20	3.7E-03				
6	Hadha	3.20	3.7E-03				
7	Hadhb	3.34	2.8E-03				
8	Acot2	2.35	0.04				
	Acot11	4.92	1.09E-09				
**Tricarboxylic acid cycle pathway**							
A	Cs	1.94	0.06	Citrate	Citrate	2.14	0.16
B	Aco2	2.35	0.04	succinate	succinate	1	0.27
C	Idh3a	2.38	0.01	fumarate	fumarate	2.05	0.14
D	Ogdh	2.65	1.5E-03	malate	malate	2.02	0.02
E	Sucla2	2.20	0.01	FAD	FAD	2.38	0.02
F	Sdha	1.88	0.07				
G	Fh1	2.43	0.01				
H	Mdh2	1.47	0.63				
I	Acss1	2.90	1.61E-03				
**Oxidative phosphorylation pathway**							
NDUFA1	Ndufa1	2.52	0.06	FMN	FMN	1.17	0.16
NDUFA10	Ndufa10	2.17	0.06	Succinate	Succinate	1	0.27
NDUFAB1	Ndufab1	2.50	0.03	Fumarate	Fumarate	2.05	0.14
NDUFB9	Ndufb9	1.89	0.07	Pi	Pi	1.59	0.02
NDUFC1	Ndufc1	1.98	0.04				
NDUFV1	Ndufv1	2.60	8.00E-03				
SDHA	Sdha	1.87	0.07				
UQCRFS1	Uqcrfs1	2.04	0.03				
UQCRC1	Uqcrc1	2.41	5.00E-03				
UQCRC2	Uqcrc2	2.02	0.04				
UQCR11	Uqcr11	2.16	0.03				
COX4	Cox4i1	2.07	0.03				
COX5A	Cox5a	2.44	0.02				
COX7A	Cox7a1	4.17	2.79E-05				
COX8	Cox8b	4.16	2.79E-05				
COX17	Cox17	1.99	0.04				
ATP5A	Atp5a	1.78	0.14				
ATP5B	Atp5b	2.30	0.03				
ATP5D	Atp5d	1.89	0.08				
PPA2	PPA2	2.05	0.03				
UCP1	Ucp1	7.5	5.07E-07				

## Discussion

This study presents the first extensive characterization and analysis of integrated molecular responses to dietary methionine restriction in key metabolic tissues in mice. A distinguishing aspect of our study is the generation of detailed transcriptomic and metabolomic signatures from the same set of samples, followed by integrative bioinformatic analysis to identify biological mechanisms that are uniquely or commonly identified by each molecular phenotype. The findings from an earlier study in rats [17] are consistent with many aspects of the current study, although there are methodological, analytical, and species differences between the studies. One key difference is that tissues were collected from non-fasted rats [17], while tissues used in the present study were from fasted mice. This may explain the differences in hepatic methionine and cysteine levels between the studies, where we found small but significant increases in methionine and cysteine levels. The study in rats reported decreases in both metabolites in the liver [17]. We speculate that compensatory mechanisms in the liver were mobilized by 4h of fasting to compensate for the reduced intake of methionine and cysteine in the MR diet. In contrast, evidence of enhanced γ-glutamyl amino acid transport was evident in both rat and mouse liver (Fig 7B), indicating induction of a common adaptive pathway to enhance glutathione synthesis in both species. Transcriptional similarities were also evident in liver and IWAT between the studies, where reduction in lipogenic capacity (liver) and enhanced oxidative activity (IWAT) were evident in both species.

### Transcriptomic and metabolomic changes in IWAT

Of the four tissues tested, we observe the maximal MR-dependent responses in IWAT, followed by liver, for both transcriptomic and metabolomic data. An inspection of differentially expressed genes in IWAT showed evidence for upregulation of fatty acid metabolism genes (*Gyk*, *Elovl3*), as well as genes (e.g., *Ucp1)* suggestive of remodeling of adipocytes to a beige phenotype in response to MR [18]. Interestingly, one of the top MR-downregulated genes in IWAT was leptin, most likely a reflection of MR-dependent activation of sympathetic outflow to IWAT in MR-treated animals [7]. At the pathway level, gene-sets related to ‘*oxidative phosphorylation’*, ‘*citrate acid cycle’*, or ‘*PPAR signaling’* showed strong evidence of transcriptional activation in IWAT that could be further integrated into broader gene regulatory networks when the gene and disease targets of several upregulated transcription factors (PPARGC1A/B, PPARA, ESRRA, NR4A3, etc.) were combined. MR-exposed tissues were thus predicted to have increased activities for mitochondrial respiration and thermogenesis and reduced activities for lipid accumulation (represented as ‘hepatic steatosis’) and intensity of carbohydrate usage (‘hypoglycemia’), probably due to better fat mobilization and oxidation (Fig 4B).

Metabolomics analysis further demonstrated a concerted up-regulation of the class of long-chain fatty acids in MR-treated IWAT, along with over-representation of significantly upregulated metabolites in pathways related to fatty acid metabolism. An integrative analysis of the transcriptomic and metabolomic changes in IWAT of MR mice (considering molecules with FDR ≤ 0.2) confirmed and highlighted an activation cascade involving three connected mechanisms viz. fatty acid transport and oxidation, tricarboxylic acid cycle, and oxidative phosphorylation (Fig 6). Three genes encoding proteins required for the transport of fatty acid from the cytosol to the mitochondrial matrix (*Cpt1*, *Slc25a20*, and *Cpt2*) were upregulated, as were fatty acyl intermediates of varying chain lengths. Genes encoding enzymes required for the subsequent process of fatty acid chain shortening via beta oxidation were also upregulated, including *Acadl*, *Acadvl*, *Hadha*, and *Hadhb*. We further observed significant upregulation of genes encoding the mitochondrial proteins *Acot2* and *Acot11*. These proteins modulate fatty acid oxidation by hydrolyzing long-chain fatty acyl-CoA into free fatty acid and CoASH. While the breakdown of fatty acyl-CoA into free fatty acids (instead of its final degradation into acetyl CoA) may seem counter-intuitive, a recent report [19] noted that overexpression of *Acot2* leads to enhanced mitochondrial fatty acid oxidation, possibly via efflux of free fatty acids from the mitochondria followed by their activation and re-entry for beta oxidation (an efflux-activation-entry circuit). This process could effectively prevent the build-up of long chain fatty acyl-CoA esters inside the mitochondria where they are known to inhibit fatty acid oxidation via substrate overload [20, 21]. Subsequent to fatty acid oxidation, one of the metabolic fates of the derived acetyl CoA is its further utilization in the tricarboxylic acid cycle.

We observed statistically significant increases in three TCA cycle intermediates (e.g., citrate, fumarate and malate). Further, with the sole exception of malate dehydrogenase, genes encoding other key enzymes of the TCA cycle were significantly upregulated, suggesting an overall expansion of TCA cycle capacity. Consistent with this scenario, we further observed significant upregulation of several components in the next linked process of electron transport/oxidative phosphorylation. Expression of several genes in each of the functional protein complexes (Complex I-V) were increased in IWAT by MR. Among the measured metabolites, we also observed increases in flavin mononucleotide (a part of respiratory chain complex I), fumarate (complex II) and inorganic phosphate (component for the generation of ATP in complex V).

Considered together, this analysis is consistent with the previously documented remodeling of IWAT that occurs after dietary MR [22]. The details provided here provide new insights into how the morphological and molecular changes expand oxidative capacity of the tissue. The MR-induced remodeling of WAT is dependent on a MR-dependent increase in sympathetic nervous system outflow to adipose tissue [7, 23]. We have recently shown that both the MR-dependent remodeling of IWAT and increase in energy expenditure are dependent on transcriptional activation and release of hepatic FGF21 by the diet [1, 4].

### Transcriptomic and metabolomic changes in liver

In contrast to the MR-driven molecular responses in IWAT, which broadly affected processes related to fuel metabolism and energy production, the consequences of MR diet on the liver transcriptome were centered on the down-regulation of pro-inflammatory signaling, and up-regulation of anti-oxidant responses. Thus, we observed a significant downregulation of KEGG pathways related to inflammatory signaling (‘*complement and coagulation cascades’*, ‘*graft vs host disease’*, etc.), whereas there was a statistically significant enrichment for genes containing the antioxidant response elements (ARE) that are activated by the transcription factor, NFE2L2 [1]. The activation of ARE-regulated genes is a well-known contributor to the regulation of cellular antioxidant defense systems, and a growing body of evidence suggests that modulation of these cytoprotective genes have profound effects on immune and inflammatory signaling [24, 25]. In the present case, the induction of ARE-regulated genes may be in response to the reduction in hepatic glutathione produced by the MR diet [6], and geared toward reversing the shortfall by enhancing glutathione synthesis [1]. The observed reduction in pro-inflammatory pathway gene expression in the liver could also be a consequence of the antioxidant response, driven by the activation of NFE2L2 responsive genes, as demonstrated in Fig 4A.

Metabolomic analysis showed the top MR-downregulated liver metabolites to largely consist of taurine-related molecules, whereas several cholate derivatives were up-regulated. Metabolomic analysis further supported the potential for a pro-oxidant state in the MR-treated liver, as evidenced by large reductions in the master anti-oxidant molecule glutathione [26], coupled with concomitant increases in opthalmate, a presumed oxidative-stress marker derived from 2-aminobutyrate and glutamate [27, 28] which were both increased in liver (Fig 7A and 7B, Fig 8, Table 3**).** Based on these observations, we envisage a scenario where reductions in methionine availability from dietary sources results in re-alignment of molecular processes to maintain hepatic methionine levels, at the expense of cysteine. Indeed, previous studies have demonstrated that the flux of compounds through the pathways highlighted in Fig 8 is largely determined by the availability of sulfur amino acids. Specifically, reactions involving enzymes with lower Michaelis constant (K_m_) are favored under scarce substrate availability. Under conditions of MR, re-methylation of methionine via homocysteine will therefore be favored over transsulfuration, as the K_m_ of Hcy transferase enzymes is two orders of magnitude lower than for cystathione synthase and cystathione gamma-lyase, which leads to the conservation of methionine at the cost of glutathione and taurine. Similarly, the K_m_ for L-cysteine tRNA synthetase (necessary for incorporation of cysteine into protein) is ten times less than that for gamma-glutamyl cysteine synthase (rate limiting step for GSH synthesis) or cysteine dioxygenase (generation of precursor for sulfate and taurine) and therefore, under conditions of limited cysteine availability, protein synthesis will be maintained and synthesis of sulfate, taurine, and GSH diminished [29, 30]. Consistent with this scenario, we observed significantly reduced levels of both taurine and glutathione (Table 3), whereas levels of methionine were slightly increased in MR-treated liver.

There were also substantial increases in levels of gamma-glutamylated amino acids, GGAA as well as their cyclized derivative, 5-oxoproline [31] and free amino acids, perhaps highlighting the importance of protein synthesis needs. Decyclization of 5-oxoproline releases glutamate, which instead of replenishing glutathione levels via ligation to cysteine, can react instead with increased levels of 2-aminobutyrate, leading to gamma glutamyl-2-amino butyrate. This intermediate further condenses with glycine to generate ophthalmic acid, via glutathione synthase. The generation of ophthalmate, at the expense of glutathione, probably reflects the consequences of increased 2-aminobutyrate and glutamate availability in MR-treated liver (Fig 8). In earlier studies, the shift from glutathione to ophthalmate production was observed under conditions of oxidative stress e.g. during paracetamol-induced hepatotoxicity [28], leading to the suggestion that ophthalmate could be a novel biomarker of hepatic glutathione depletion [32]. According to this scenario, the very large increase in ophthalmate levels (>35-fold), coupled to a significant lowering of GSH (~4-fold reduction, from 250 nmol/g) that was observed in the current study would be consistent with a net pro-oxidant environment in MR-treated liver. In contrast to the liver, an adequate GSH driven anti-oxidant response appears intact in MR IWAT where levels of both reduced and oxidized forms of glutathione are increased and no statistically significant induction of ophthalmate is noted (Table 3). Faced with a pro-oxidant milieu, the MR liver is predicted to mobilize anti-oxidant responses, perhaps via activation of the NFE2L2 transcription factor, as predicted by IPA analysis of the transcriptome (Fig 4A). In response to oxidative stress, NFE2L2 is known to undergo nuclear translocation and bind to the antioxidant response elements (AREs) of a number of anti-oxidant genes, resulting in their transcriptional activation [33]. Thus, the observed transcriptional induction of the 21 NFE2L2 target genes strongly suggests the activation of an anti-oxidant program in MR liver. Upregulation of the anti-oxidant defense response may also help explain the observed reduction in hepatic pro-inflammatory signaling pathways observed in our study; this is consistent with earlier reports linking anti-oxidant responses to amelioration of hepatic pro-inflammatory signaling [34, 35]. Together, our transcriptional and metabolomics data indicate that the liver preserves methionine at the expense of cysteine, glutathione, and taurine in mice consuming the MR diet. The evidence suggests that the perception of a glutathione reduction may be the key signal that activates the NFE2L2 transcriptional program that increases a cadre of genes that function to correct the reduction in hepatic glutathione [1]. These findings point to cysteine and its downstream product, glutathione, as key signaling molecules linking dietary MR to its metabolic phenotype. This conclusion is supported by recent findings that addition of small amounts of cysteine to the MR diet reverses the reduction of liver glutathione, reverses the activation ARE genes, and reverses the induction of hepatic FGF21 and most components of the MR phenotype [1]. The present studies extend our understanding of these processes by providing new insights into how restricting dietary methionine affects sulfur amino acid metabolism in the liver.

## Supporting information

S1 ARRIVE Checklist ARRIVE guidelines checklist. (PDF)

S1 Table Top differentially expressed genes in MR treated vs. Control tissues. Genes at false discovery rate (FDR) <5% are shown for IWAT, liver and BAT. No genes were identified at FDR<5% in muscle.
(XLS)

S2 Table Top KEGG pathways identified by GSEA for MR-treated tissues. Pathways with a false discovery rate (q-value) < 10% are shown. Column descriptors are as follows: Name, Name of KEGG pathway; Size, Number of genes contained in pathway; NES, Normalized enrichment score, a GSEA derived metric normalized for pathway size and reflecting the degree of pathway enrichment; NOM p-val, nominal GSEA p-value derived from gene-label permutations; FDR q-value, false discovery rate estimate; Direction, direction of pathway enrichment in MR or control samples; Tissue, tissue where pathway was identified.
(XLS)

S3 Table Predicted changes in upstream regulators by dietary methionine restriction. Analysis was carried out in Ingenuity and regulators with information on both z-scores and overlap p-values are reported for each tissue. Column 1, name of regulator (transcription factor); column 2, changes in expression of regulator upon methionine restriction, if any; column 3, type of regulator; column 4, predicted activation state of regulator (activation or inhibition); column 5, activation z-score; column 6, p-value for overlap between differentially regulated genes and regulator target genes; column 7, regulator target genes that are differentially expressed in dataset; column 8, tissue of interest.
(XLS)

S4 Table Metabolite expression summaries by tissues. Column 1, biochemical name of metabolite; column 2, ratio of metabolite expression levels in MR vs Control samples; column 3, nominal significance of differential expression; column 4, adjusted p-values; column 5, biochemical pathway; column 6, tissue examined.
(XLS)

## Abbreviations

MR: Methionine Restriction
GSEA: Gene Set Enrichment Analysis
ORA: Over Representation Analysis
IPA: Ingenuity Pathway Analysis
KEGG: Kyoto Encyclopedia of Genes and Genomes
FDR: False Discovery Rate
RIN: RNA Integrity Number
BRB: Biometric Research Branch
